# Intraperitoneally infused human mesenchymal stem cells form aggregates with mouse immune cells and attach to peritoneal organs

**DOI:** 10.1186/s13287-016-0284-5

**Published:** 2016-02-10

**Authors:** Nikolay Bazhanov, Joni H. Ylostalo, Thomas J. Bartosh, April Tiblow, Arezoo Mohammadipoor, Andrea Foskett, Darwin J. Prockop

**Affiliations:** Institute for Regenerative Medicine, Texas A&M Health Science Center College of Medicine at Scott & White, 5701 Airport Rd., Temple, TX 76502 USA

**Keywords:** MSC, Mesenchymal stem cells, Intraperitoneal injections, Cell tracking, Cytokines

## Abstract

**Background:**

Mesenchymal stem/progenitor cells (MSC) have shown beneficial effects in many models of disease in part by modulating excessive inflammatory and immune responses. Frequently the beneficial effects of MSC persist long after their disappearance from host tissues, suggesting that MSC interact with intermediate cells in the host that relay or amplify their effects. The cells have usually been injected intravenously, but beneficial effects have also been reported with intraperitoneal (IP) injection of MSC. However the fate of IP injection of MSC has not been examined.

**Methods:**

The fate of the human MSC injected IP into immune-competent mice was studied. In vivo imaging was used to track green fluorescent protein-labeled MSC in the peritoneal cavity. In addition, their retention in peritoneal tissues was measured by real-time polymerase chain reaction for human GAPDH mRNA. To describe the effects of human MSC on the immune system of the peritoneum, the peritoneal lavage, omentum, lymph nodes and mesenteric tissues were collected. Flow cytometry was used to evaluate the immune cell populations, while cytokine/chemokine production was measured by real-time polymerase chain reaction and enzyme-linked immunosorbent assay. Challenge with lipopolysaccharide at 3 days after the administration of MSC was used to evaluate the preconditioning of the immune system.

**Results:**

Within 20 min, single MSC were no longer detected in peritoneal lavage fluid. Instead they were recovered as aggregates of varying size that contained mouse macrophages and a few B220+ lymphocytes. After 1 day, most of the aggregates containing live MSC were attached to sites throughout the peritoneal cavity including the omentum and mesentery. Less than 0.05 % of the live injected cells were detected in the spleen and jejunal lymph nodes. In all locations, MSC colocalized with mouse macrophages and B220+ lymphocytes. Attachment to the omentum and mesentery was accompanied by the recruitment of immune cells and changes in the production of a series of mouse cytokines. A similar increase in mouse cytokines in the peritoneum was seen after IP injections of human fibroblasts.

**Conclusions:**

IP injected human MSC rapidly formed aggregates with mouse macrophages and B220+ lymphocytes and attached to the walls of the peritoneal cavity. The formation of the aggregates probably limits access of the cells to the systemic circulation.

**Electronic supplementary material:**

The online version of this article (doi:10.1186/s13287-016-0284-5) contains supplementary material, which is available to authorized users.

## Background

Human mesenchymal stem/progenitor cells (MSC) from bone marrow, adipose tissues, placenta, umbilical cord and other tissues are currently being administered to large numbers of patients. Over 100 clinical trials with MSC have been registered [1], and several have reached phase II or III stage of development [2]. In essentially all the clinical trials, and in most experiments in animal models, the cells were administered by intravenous (IV) injection. At the same time, there has also been interest in administration by injection into the peritoneum (intraperitoneal; IP) [3–5]. For example, it has been previously shown that MSC reach the lungs after IP injection [6]. One advantage of IP injection is that, because of slower rate of their migration from the peritoneal cavity, IP injection could potentially avoid rapid embolization of the lung vasculature that can be lethal [7]. Also, IP injection allows for the administration of larger numbers of cells. In addition, in direct comparisons with IV administrations, IP injected MSC were shown to have comparable or even more profound effects in experimental autoimmune encephalitis [8], twitcher mice with globoid cell leukodystrophy [9], mice with cisplatin-induced renal injury [10], mice with experimental intestinal colitis [11], mice with sterile inflammation of the cornea [12], and mice with zymosan-induced peritonitis [13]. The beneficial effects of MSC in these and other disease models have been linked to the ability of the cells to modify both the innate and acquired immune systems [14, 15].

The peritoneal cavity provides an unusual setting for immune interactions because it is primed to respond rapidly to bacteria that may be released by intestinal spillage [16], allergens such as worms that enter the gastrointestinal tract, or tumor metastases [17]. The rapidity of the response is obviously critical. It is mediated by at least two special classes of cells. One special class is the B lymphocytes, particularly B-1b lymphocytes, found in the peritoneal cavity and other sites such as the omentum that contain an unusual repertoire of receptors that allow them to mount an immediate antibody response to bacteria without being part of the adaptive immune system [18]. The second special class is small cells that are found in the mesentery and some other locations in the body and that are triggered by worms and other allergens to mount a rapid T-helper (Th)2 response. The cells are lineage-negative and referred to as nuocytes among several other terms [19–23]. The cells generate an innate Th2 response characterized by secretion of interleukin (IL)-4 that facilitates production of immunoglobulin (Ig)G1 and IgE, and by secretion of IL-13 that contributes to epithelial hypertrophy and recruitment of eosinophils, characteristics of allergic responses [19, 22].

The peritoneal cavity is also an unusual setting for entry into the immune system because it participates in dynamic exchange of the fluids and cells between the cavity and both the lymphatic system and the general circulation. The exchange occurs through three or four portals that breach the tight layer of basement membrane and mesothelial cells that forms the peritoneal membrane and that covers all the organs and the walls of the abdomen: (a) “milky spots” that are opaque, highly vascularized clusters of cells on the surface of the omentum, the fatty organ that straddles the spleen, stomach and other organs of the upper abdomen in mice but covers a much more extensive area in humans; (b) the draining lymphatic system contained in the stalk of mesenteric arteries and veins that supplies the gastrointestinal tract and in the walls of the peritoneal cavity; (c) punctate regions called lymphatic stomata on the underside of the diaphragm; and (d) the newly discovered small cells in the mesentery and other tissues that mount a rapid Th2 response [19–23]. The milky spots of the omentum [23, 24] contain clusters of macrophages and B1 lymphocytes that are similar to the free-floating macrophages and B1 lymphocytes of the peritoneal cavity. The milky spots readily exchange particulate matter and cells between the peritoneal cavity and the general circulation. They may [25] or may not [24] contain resident dendritic cells that can activate lymphocytes, but they can recruit T cells activated at other sites. The draining lymphatics of the mesenteric stalk and peritoneal wall absorb particles from the gastrointestinal tract, and both particles and cells from the peritoneal cavity. The lymphatic stomata on the underside of the diaphragm consist of a thin layer of cuboidal cells that overlay large lacunae of lymphatic vessels [26, 27]. The lacunae drain primarily into mediastinal lymph nodes and then to the thoracic duct and eventually the left subclavian vein.

In exploring the fate of MSC injected into the peritoneal cavity, several groups of investigators observed engraftment of the cells not only into the peritoneum but also into multiple distal organs [28, 29]. However, the route the cells traveled was not defined. In initial efforts to follow the fate of human MSC injected into the peritoneum, we recently reported [30] that some of the MSC aggregated and compacted into spheres similar to the spheres formed after the cells were cultured in hanging drops. Here we report that, after IP administration of MSC into immunocompetent mice, the cells rapidly formed aggregates with macrophages and a few B220+ cells. The aggregates were initially recovered in the peritoneal lavage fluid but then they slowly attached to milky spots in the omentum and to regions of the mesentery. Apparently, because of the large size of the aggregates, few of the MSC appeared in mesenteric lymphatic nodes, a route previously shown to be favored by IP injected splenocytes [31]. The MSC disappeared in less than a week but prompted recruitment of cells to the omentum and peritoneal cavity that secreted both pro- and anti-immune mouse cytokines. Similar increases in mouse cytokines in the peritoneum were seen after IP administration of human fibroblasts.

## Methods

### Human mesenchymal stem/stromal cell culture

Human MSCs were obtained from normal, healthy donors with informed consent under Scott & White and Texas A&M Institutional Review Boards approved procedures. Passage 1 wild-type MSC and passage 4 green fluorescent protein-expressing MSC (GFP-MSC) were obtained as frozen vials from the Center for the Preparation and Distribution of Adult Stem Cells [32]. Human MSC (donors 7075, 7068, and 7032) were isolated from bone marrow aspirates and cultured as previously described [33]. Human GFP-MSCs were generated from passage 0 wild-type human MSCs using lentiviral vector containing humanized recombinant form of green fluorescent protein under the control of chicken beta-actin promoter and cytomegalovirus enhancer WPT-CAG-hrGFP (Stratagene) pseudotyped with VSVG envelope in the presence of polybrene (Sigma). The resultant population was purified using fluorescent activated cell-sorting (FACSVantage; Becton Dickinson) and expanded for three more passages. The cells met the standard criteria for MSC, including profile of surface epitopes, CFU values and trilinage differentiation. In vitro growth, differentiation, clonogenicity and epitope markers of MSC preparations are summarized in Additional file 1 (Table S1) according to Reger and Prockop [34]. For the experiments described here, a frozen vial of about 1 million passage 1 MSC was plated in complete culture medium (CCM) consisting of α-minimum essential medium (αMEM; Gibco, Grand Island, NY), 17 % fetal bovine serum (FBS; Atlanta Biologicals, Lawrenceville, GA), 100 units/ml penicillin (Gibco), 100 mg/ml streptomycin (Gibco), and 2 mM L-glutamine (Gibco) on a 152 cm^2^ culture dish (Corning, Acton, MA) for 24 h. Cells were washed with phosphate-buffered saline (PBS) and the adherent viable cells were harvested using 0.25 % trypsin and 1 mM ethylenediaminetetraacetic acid (EDTA; Gibco) for 3–4 min at 37 °C, reseeded at 100–200 cells/cm^2^ in CCM and incubated for 6–7 days (with medium change every 2–3 days) before freezing in αMEM containing 30 % FBS and 5 % dimethylsulfoxide (Sigma, St. Louis, MO). For the in vivo experiments, the cells were recovered as passage 2 (MSC) and passage 5 (GFP-MSC), cultured for 24 h, and re-plated at 100–200 cells/cm^2^. The cells were cultured for 6–7 days and harvested with trypsin-EDTA, washed once in Hanks Balanced Salt Solution (HBSS; Lonza, Basel, Switzerland) containing 0.1 % FBS and then again washed with serum-free HBSS. The cells were finally reconstituted at 1 × 10^4^ cells/μl to a total amount of 0.3–3 × 10^6^ cells in serum-free HBSS and kept on ice for the injections.

### Human adult dermal fibroblast culture

Human adult dermal fibroblasts (hDFs) were obtained from American Type Culture Collection (ATCC). Frozen vials of the cells were thawed and plated on adherent T-175 flasks (Corning) in CCM for 24 h. After medium change, the cells were expanded until approximately 70–90 % confluent. Cells were harvested with trypsin/EDTA for 5 min at 37 °C and re-plated at 1000–3000 cells/cm^2^ for expansion. Medium was changed every 2–4 days and cells were harvested at 70–90 % confluence for assays.

### Single GFP cell measurements in peritoneal cavity

All animal procedures were approved by the Animal Care and Use Committee of Texas A&M University Health Science Center and in accordance with guidelines set forth by the National Institutes of Health.

Male 6-week-old BALB/c mice (Jackson Laboratories, West Grove, PA) were injected IP with 3 × 10^6^ GFP-MSC suspended in 300 μl HBSS. At 1 min, 20 min, 4 h and 72 h after the administration of MSC, mice were euthanized by cervical dislocation under deep anesthesia and peritoneal lavage was collected by injecting 5 ml of PBS into the peritoneal cavity followed by harvesting the lavage with a pipette. Ten microliters of the lavage were then placed directly in Neubauer hemocytometer in duplicate, and fluorescent and brightfield images were taken using inverted Nikon Eclipse Ti-S microscope (Nikon). The “Analyze Particles” plugin for ImageJ software was used to count total and GFP-positive cells.

### Real-time PCR for assay of human cell number

To estimate the total number of human cells in the omentum, jejunal lymph nodes, mesentery, spleen and peritoneal lavage, human glyceraldehyde 3-phosphate dehydrogenase (GAPDH) expression was measured [7, 12]. Standard curves were prepared by combining known amounts of MSC with tissues and peritoneal resident cells from naïve BALB/c mice. Total RNA was isolated using RNeasy Lipid Tissue Mini Kit (Qiagen) with DNA digestion step using DNAse (Qiagen). Isolated RNA was quantified using Nanodrop specrophotometer (Thermo Fisher Scientific) and 0.3–2 μg was converted to cDNA with High-Capacity cDNA RT Kit (Applied Biosystems, Foster City, CA). Real-time polymerase chain reaction (PCR) for eukaryotic 18S ribosomal RNA and human GAPDH was performed using Taqman Gene Expression Assays (Applied Biosystems) and Taqman Fast Master Mix (Applied Biosystems) in triplicate using 20 ng cDNA in 20 μl reaction. Real-time PCR reaction was performed with CFX96 Real-Time PCR Detection System (Biorad, Hercules, CA) by incubating the reactions at 95 °C for 20 s followed by 40 cycles of 95 °C for 1 s and 60 °C for 20 s. Delta Ct values between human GAPDH and 18S were calculated and used to generate standard curves (Additional file 2: Figure S1A). Microsoft Excel was used to perform nonlinear regression fit and obtain formulas for human cell number calculations (Additional file 3: Table S2). Delta Ct values between human GAPDH and eukaryotic 18S were then obtained for samples from MSC-injected animals and used to calculate total cell numbers based on formulas provided in Additional file 3 (Table S2).

### Preparation of dead MSC

A total of 3 × 10^6^ MSC in HBSS at 1 × 10^4^ cells/μl were frozen by immersion into liquid nitrogen followed by thawing at 37 °C. These steps were repeated three times. The cells were then placed on ice for injections. The viability of the cells was assayed by trypan blue staining (Gibco), and more than 99 % of the cells were positive for uptake of the dye.

### Whole tissue, blood, and peritoneal lavage fluid collection

Experimental and control mice were euthanized by cervical dislocation under deep anesthesia. The rib cage was opened and blood was collected from the right ventricle of the heart and placed in a tube containing clotting activators (Terumo Medical Corporation, Somerset, NJ) for 20 min and then stored on ice until further processing. Serum was separated by centrifugation at 1500 × g for 10 min and stored at –80 °C until further analysis. To obtain lavage fluid for assays of cytokines, 1 ml of PBS containing Halt Protease inhibitor Cocktail (Thermo) was injected IP, followed by gentle massaging and collection of fluid with a sterile pipette. Complete harvest of the peritoneal cells was obtained by another injection of PBS (5 ml) followed by lavage harvest. The cell suspensions from both collections were separately centrifuged at 500 × g for 10 min at 4 °C. Supernatant from the first wash was further clarified by centrifugation at 10,000 × g for 10 min at 4 °C, aliquoted and stored for further cytokine analysis at –80 °C. The supernatant from the second wash was discarded and cell pellets were combined and homogenized with Trizol lysis buffer (Qiagen, Germany), flash frozen in liquid nitrogen, and stored at –80 °C until further processing. Mouse omentum, jejunal lymph nodes, mesentery, and spleen were collected and either flash-frozen in liquid nitrogen for later RNA isolation, or placed in sterile HBSS for enzymatic digestion.

### In vivo tracking of GFP-MSC

We used male BALB/c mice that were 6–8 weeks of age and housed on a 12-h light/dark cycle. A total of 2–3 × 10^6^ GFP-MSC in 300 μl HBSS were injected IP with a 28G needle under brief isoflurane anesthesia. At 4, 24, 72 and 168 h, the animals were euthanized and their peritoneal cavities exposed. For the visualization of GFP-MSC in omentum, the organs were excised from peritoneal cavities and placed on a plate with a white background. Lumina IVIS II System (Perkin Elmer, Waltham, MA) was used to obtain fluorescent images of the whole peritoneal cavity and individual omenta. Living Image Software version 4.1 (Perkin Elmer) was used for GFP fluorescence analysis. To aid in visualization, the GFP signal was pseudocolored by applying logarithmic grayscale to the whole peritoneal cavity image or by applying reverse logarithmic grayscale to the images of the omenta.

### Immunofluorescence of free-floating aggregates

Free-floating GFP+ aggregates were picked with tweezers from the peritoneal cavity 24 h after the IP injection of GFP-MSC. The aggregates were washed twice with PBS, and fixed with 2 % paraformaldehyde (Affymetrix, Santa Clara, CA) in PBS for 2 h. The fixed aggregates were washed twice with PBS, centrifuged at 500 × g for 5 min, and incubated at 4 °C overnight in 1 ml 30 % sucrose (Sigma) in 0.1 M phosphate buffer (Sigma). After 24 h, the aggregates were collected in 800 μl of 50 % optimal cutting temperature compound (OCT; Sakura Finetek, Torrance, CA) and transferred into a histology mold. The mold was frozen in isopentane (Sigma), chilled by liquid nitrogen and stored at –80 °C. Cryosections (6 μm) were prepared with a Microm HM560 cryostat and incubated for 20 min at room temperature before storing at –80 °C. For the immunofluorescent staining, sections were equilibrated to room temperature, fixed again for 10 min in 2 % paraformaldehyde and washed twice in Tris-Buffered Saline with Tween (TBST; Cell Signaling, Beverly, MA). Nonspecific antibody binding was blocked by incubating samples for 45 min in TBST supplemented with 5 % bovine serum albumin (BSA; Thermo Fisher Scientific, Whaltman, MA) and 5 % normal serum (Thermo). Following two washes in TBST, samples were incubated for 24 h at +4 °C with primary antibodies to mouse CD11b (catalog #550282; BD Pharmingen, Frankling Lakes, NJ) at 1:100 dilution in blocking solution, or isotype control antibody. Sections were washed three times in TBST, incubated for 1 h at room temperature with anti-rat Alexa-594 conjugated secondary antibodies (Life Technologies, Grand island, NY) at 2 μg/ml in blocking solution, then counterstained with 0.5 μg/ml of 4,6-diamidino-2-phenylindole (DAPI; Sigma) in PBS for 10 min. The sections were washed three times in TBST and mounted in Prolong Gold antifade reagent (Life Technologies) overnight.

### Whole mount immunofluorescence of aggregates in the mesentery and omentum

Whole omenta or portions of mesentery with visible GFP+ clusters were removed from the peritoneal cavity 24 h after GFP-MSC injection, washed twice with PBS, and fixed with 2 % paraformaldehyde in PBS for 2 h. Fixed tissues were washed twice with PBS and blocked by incubation in TBST with 5 % BSA and 5 % normal serum for 45 min at room temperature. Samples were washed twice with TBST, and incubated for 24 h at +4 °C with primary antibody to mouse CD45R (catalog #550286; BD Pharmingen) or isotype control antibody at 1:100 dilution in blocking solution. Tissues were then washed three times in TBST, incubated for 1 h at room temperature with anti-rat Alexa-594 conjugated secondary antibodies (Life Technologies) at 2 μg/ml in blocking solution, then counterstained with 0.5 μg/ml DAPI in PBS for 10 min. The tissues were placed on a glass slide and covered with a coverslip.

### Immunofluorescent image acquisition and manipulation

Low magnification (1–2×) bright-field and GFP images were captured with Nikon Digital Sight DS-2Mv camera attached to a SMZ800 dissecting microscope (Nikon, Japan). Illumatool Bright Light Systems LT 9900 (Lightools Research) with GFP filter set (470 nm excitation and 515 nm emission) was placed under the objective of the microscope to visualize GFP fluorescence. High magnification images were obtained using Nikon Eclipse 80i upright microscope and processed using NiS Elements AR3.0 software (Nikon, Japan). All image manipulations (merging, brightness and contrast adjustment) were performed using ImageJ Version 1.49a (NIH, US).

### Real-time PCR for mouse cytokines

Mouse omenta, jejunal lymph nodes, mesentery, spleen and cell pellet that included MSC aggregates from peritoneal lavage were obtained as described above and flash-frozen in liquid nitrogen. The tissues were homogenized in Trizol lysis buffer (Qiagen, Germany) and total RNA was isolated using RNeasy Lipid Tissue Mini Kit (Qiagen) with DNA digestion step using DNAse (Qiagen). Isolated RNA was quantified using Nanodrop specrophotometer (Thermo Fisher Scientific) and 0.3–2 μg was converted to cDNA with High-Capacity cDNA RT Kit (Applied Biosystems, Foster City, CA). Real-time PCR for mouse *Il10, Il13, Ifng* and *Ptgs2* was performed using Taqman Gene Expression Assays (Applied Biosystems) and Taqman Fast Master Mix (Applied Biosystems) in triplicate using 20 ng cDNA in 20 μl reaction. Real-time PCR reaction was performed with CFX96 Real-Time PCR Detection System (Biorad, Hercules, CA) by incubating the reactions at 95 °C for 20 s followed by 40 cycles of 95 °C for 1 s and 60 °C for 20 s. Calculated delta-Ct values between gene of interest and *Gapdh* were used to obtain relative expression values (2^–ΔCt^).

### Assays of cells in the omentum

Omenta were excised from the animals and placed in HBSS with calcium and magnesium containing 0.8 mg/ml dispase/collagenase, 0.2 mg/ml collagenase P and 0.1 mg/ml DNAse I (Roche Molecular Diagnostics, USA, Pleasanton, CA). The omenta were minced and incubated at 37 °C for 60 min with gentle pipetting every 15 min. The digest was then diluted 10 times with calcium and magnesium-free HBSS, strained via a 70-μm nylon mesh and centrifuged at 500 × g for 5 min at room temperature. The supernatant was discarded and the cell pellet was resuspended in calcium- and magnesium-free HBSS, centrifuged again, and resuspended in HBSS containing 2 % BSA, followed by cell counting with hemocytometers. The cells were then incubated for 10 min at 4 °C with anti-CD16/32 antibody at a concentration 0.5 μg per 1 × 10^6^ cells in 100 μl of PBS containing 1 % BSA (eBioscience) to block nonspecific binding to Fc-receptors. After one wash with PBS supplemented with 1 % BSA, the cells were incubated for 20 min at room temperature with fluorescently conjugated antibody against mouse F4/80 (eBioscience), Ly6G, CD19, CD3, CD8, and CD45R (BD Pharmingen). The antibodies were used at a concentration of 1 μg per 1 × 10^6^ cells in 100 μl PBS supplemented with 1 % BSA. Isotype-matching antibodies at similar concentrations obtained from the same manufacturers and single-color labeling were used as controls for the specificity of labeling. After two washes in PBS, the cells were resuspended in PBS supplemented with 1 % BSA and analyzed by flow cytometer (Model FC500; Beckman Coulter, USA, Brea, CA) to determine macrophage (F4/80-positive), neutrophil (Ly-6G-positive), T-cell (CD3- or CD8-positive) and B-cell (CD19- or CD45R-positive) populations.

### Assays for mouse secreted cytokines

Mouse IL-6, IL-10, IL-12p70, interferon (IFN)γ, monocyte chemotactic protein-1 (MCP1), chemokine (C-X-C motif) ligand 1 (CXCL1), transforming growth factor (TGF)β1, tumor necrosis factor (TNF)-α and prostaglandin (PG)E_2_, in peritoneal lavage and/or serum, were assayed with commercial enzyme-linked immunosorbent assay (ELISA) kits (R&D Systems Inc, Minneapolis, MN). Mouse IL-13 was assayed with commercial ELISA kit from Life Technologies. For all assays, optical density was determined on a plate reader (FLUOstar Omega; BMG Labtech, Germany) at an absorbency of 450 nm with wavelength correction at 540 nm for the optical imperfections on the plate.

### Lipopolysaccharide injury model in mice

Male 6-week-old BALB/c mice were injected IP with 3 × 10^6^ MSC or dead MSC, prepared as described above. Sterile HBSS injections were used as controls. At 3 days after the MSC delivery, the animals were injected with lipopolysaccharide (LPS; Sigma) at 0.1 mg/kg body weight via the tail vein. Three hours after the administration of LPS, tissues were harvested and lavage fluid collected.

### Data analysis and presentation

When comparing two groups, the unpaired *t*-test was used, whereas one-way analysis of variance (ANOVA) with Bonferroni’s post-hoc analysis was used in multiple comparisons. One-way ANOVA with Dunett’s post-hoc analysis was used to compare groups to a control. Null hypotheses were rejected at *P* values less than 0.05. All statistical analyses and chart preparations were performed with GraphPad Prism 6 software (GraphPad Software, Inc., USA, La Jolla, CA). Curve fitting was performed using Microsoft Excel Software. Images and charts were combined and annotated using Inkscape Software.

## Results

### IP administration of different human MSC preparations and fibroblasts

**Fig. 1:**
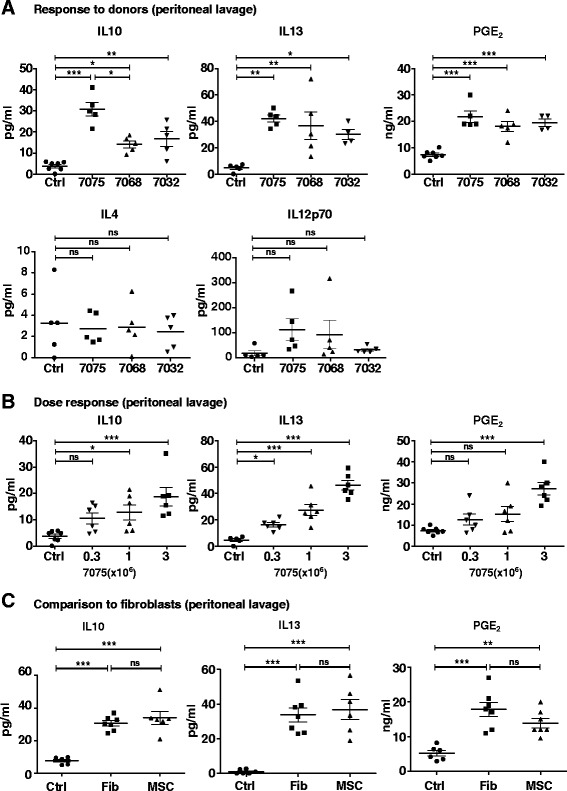
Effect of mesenchymal stem cells or fibroblast administration on cytokine production in the peritoneum. Mouse IL-10, IL-13, PGE_2,_ IL-4 and IL-12p70 were assayed at 72 h after IP MSC or HBSS (*Ctrl*) administration into BALB/c mice. **a** Mouse cytokine production in the peritoneum in response to three donors. **b** Peritoneal levels of mouse cytokines after administration of various doses of MSC. **c** Mouse cytokine production in the peritoneum in response to fibroblasts (*Fib*) compared to HBSS (*Ctrl*) or mesenchymal stem cell (*MSC*) administration. *P < 0.05, **P < 0.01, ***P < 0.001, compared to controls. *IL* interleukin, *PGE*_*2*_ prostaglandin E_2_

Different preparations of MSC from human bone marrow vary in their properties and biological activities, a feature loosely referred to as donor variation [35, 36]. Therefore we administered three different preparations of MSC IP in mice and compared their effects on the levels of mouse cytokines in peritoneal lavage fluid. All three increased the levels of IL-10, IL-13 and PGE_2_ but not IL-4 or IL-12p70 (Fig. 1a). Because MSC designated as Donor 7075 had the most consistent effects, we employed the Donor 7075 in further experiments. The IP administration of Donor 7075 MSC demonstrated a progressive increase in the levels of IL-10, IL-13 and PGE_2_ with 0.3, 1 and 3 × 10^6^ (Fig. 1b). In addition, an increase in systemic IL-13 and IL-10 was observed after IP administration of MSC donor 7075 (Additional file 4: Figure S2). Therefore we employed administration of 3 × 10^6^ MSC in further experiments. Similar increases in the three responding cytokines (IL-10, IL-13 and PGE_2_) were observed after IP administration of human skin fibroblasts (Fig. 1c). Therefore the effects were not specific to administration of MSC.

### Fate of human MSC in the peritoneal cavity

**Fig. 2:**
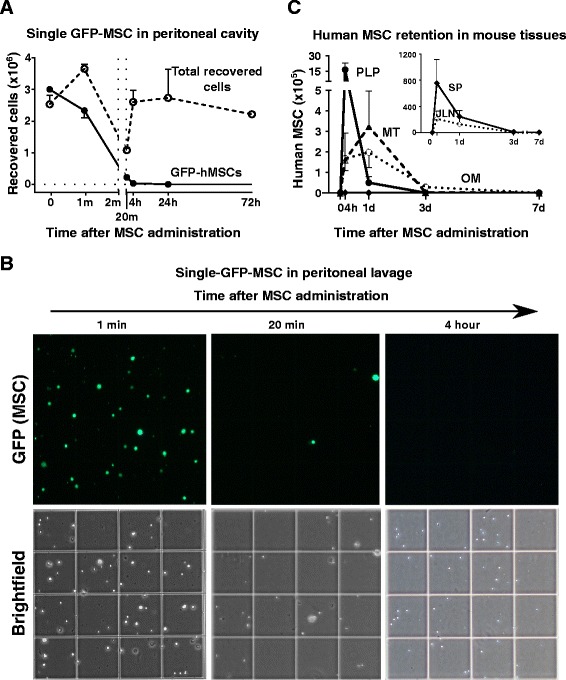
Fate of the IP injected mesenchymal stem cells in the peritoneal cavity. **a** Changes in the numbers of single GFP-positive cells and total single cells in the peritoneal cavity after intraperitoneal administration of GFP-MSC into BALB/c mice. The cells were recovered by a 5 ml wash of the peritoneal cavity with PBS. The resulting lavage was gently mixed and the single cells counted in a hemocytometer (n = 4, mean ± SEM). **b** Fluorescent images of single GFP-MSC in the hemocytometer chamber; magnification 20×. **c** Total number of human cells in pellets of peritoneal lavage (*PLP*), random sampling of about 25 mg or about 10 % of mesentery (*MT*), omentum (*OM*), jejunal lymph nodes (*JLN*) and spleen (*SP*). To collect PLP, peritoneal lavage was collected by washing the peritoneal cavity first with 1 ml and then with 5 ml of sterile PBS. The two samples of lavage were centrifuged at 500 × g for 5 min and the pellets were combined. Human cells in the samples were assayed by real-time RT-PCR for human GAPDH mRNA normalized to total eukaryotic 18S. Data points represent arithmetic means ± SEM, n = 3–5. Insert indicates human cell numbers in the spleen and jejunal lymph nodes. Note dramatic differences in cell numbers between the insert and the large panel. *GFP* green fluorescent protein, *MSC* mesenchymal stem cells

To study the fate of MSC injected into the peritoneal cavity, GFP-labeled human MSC were used. To detect single GFP-labeled cells in the peritoneal cavity, 5 ml of PBS was injected IP and the harvested lavage fluid was examined in a hemocytometer (Fig. 2a and b). One minute after injection, 75 % of the injected GFP-labeled cells were accounted for (Fig. 2). Surprisingly, the number fell to less than 10 % after 20 min. At the same time, there was a marked decrease in the total number of mouse cells in the lavage fluid, apparently because macrophages and some lymphocytes in the peritoneal cavity had aggregated with the MSC [37]. The number of murine single cells increased by 4 h and it continued to increase for at least 72 h (Additional file 2: Figure S1B).

Aggregation of the injected MSC was demonstrated by assays of the pellet obtained by low-speed centrifugation (500 × g for 10 min) of the peritoneal lavage. Four hours after injection of the MSC, assays by real-time PCR for human GAPDH mRNA demonstrated that about 50 % of the injected human cells were recovered in the pellet (Fig. 2c; Additional file 2: Figure S1A; Additional file 3: Table S2; and Additional file 5: Table S3). However, at 1 day, only about 1 % of the injected cells were recovered in the pellet of the peritoneal lavage (Fig. 2c), apparently because the aggregates had attached to the mesentery, omentum and other surfaces of the peritoneal cavity. Of special interest was that less than 1500, or 0.05 %, of the injected 3 × 10^6^ cells were found in the spleen and jejunal lymph nodes at any of the time points examined (insert in Fig. 2c).

**Fig. 3:**
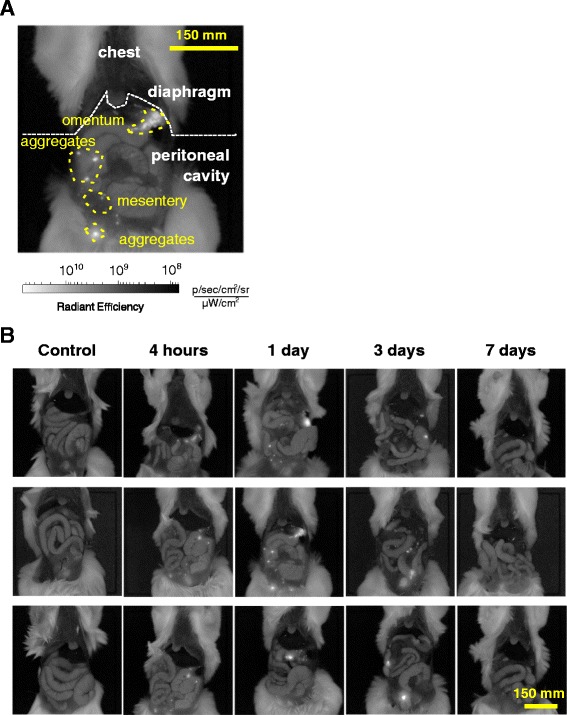
In vivo fluorescent imaging of GFP-MSC in the peritoneal cavity. **a** Depiction of GFP-MSC distribution in the peritoneal cavity of BALB/c mice 24 h after IP administration. *Yellow dashed lines* outline GFP-positive areas (*bright white*) corresponding to the omentum, aggregates and portions of mesentery. **b** Representative images of the peritoneum of BALB/c mice injected IP with GFP-MSC and followed by in vivo fluorescent imaging. *Bright white areas* correspond to the areas of GFP-positive cells. Logarithmic grayscale was applied to panels (**a**) and (**b**) to help in visualization of the GFP signal

To examine the fate of the injected cells further, we injected GFP-MSC IP and opened the peritoneal cavity to follow the distribution of the cells by in vivo fluorescent imaging (Fig. 3). As expected from the assays of the lavage pellet (Fig. 2c), numerous aggregates of GFP-positive cells were found as free-floating in the peritoneal cavity at 4 h (Fig. 3a and b). At 1 day, the GFP-positive aggregates appeared to have increased in size and become attached to multiple surfaces within the peritoneal cavity. Many were easily detached by flushing with PBS or lifting with tweezers. However, the aggregates that attached to the omentum and to sites within the mesentery were firmly adherent and appeared to be embedded within the tissues. At 3 days, there was a decrease in GFP-labeled aggregates and at 7 days they were no longer detected.

**Fig. 4:**
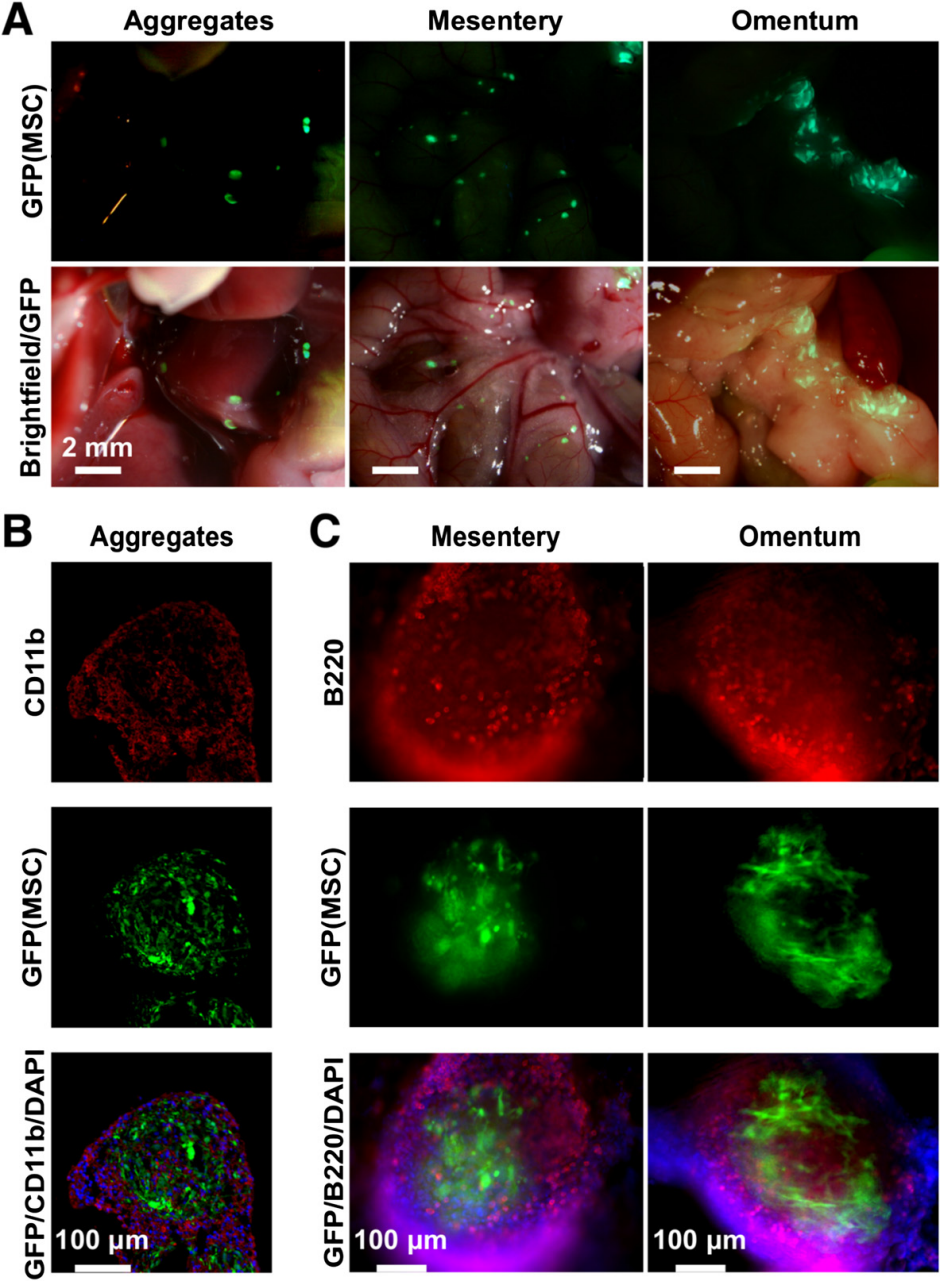
Colocalization of human GFP-MSC with immune cells in the peritoneal cavity. Representative immunofluorescent images of human GFP-MSC in free-floating aggregates, and aggregates attached to the mesentery and omentum 24 h after their IP administration. **a** In vivo low-magnification (1.5×) images. **b** GFP-positive aggregates as shown in (**a**) were obtained from the peritoneal cavity, cryosectioned and stained with CD11b antibody (magnification 20×). **c** GFP-positive aggregates attached to the mesentery and omentum were isolated with tweezers, fixed, labeled with B220 antibody, and examined as whole mounts (magnification 20×). Representative images from three mice. *DAPI* 4′,6-Diamidino-2-phenylindole; *GFP* green fluorescent protein, *MSC* mesenchymal stem cells

To examine the composition of the aggregates, GFP-MSC were injected IP, and GFP-positive aggregates in the peritoneal lavage, mesenteric sites, and the omentum were isolated after 24 h and examined (Fig. 4a). Immunofluorescent images of sections of aggregates from the peritoneal lavage demonstrated that they were rich in cells positive for CD11b + (Fig. 4b), a marker for macrophages. Immunohistochemistry of whole mounts of aggregates from mesenteric sites and the omentum demonstrated that they were rich in B220+ cells (Fig. 4c), a marker expressed in various immune cells including B1 lymphocytes that are the predominant lymphocytes of the peritoneal cavity and the omentum, macrophages, T cells and dendritic cells. The micrographs and a z-stack video of an aggregate from the omentum demonstrated that the GFP-MSC were at the center of the aggregates without any specific orientation (Online Additional file 6: Video S1).

### The MSC aggregates in the omentum recruit macrophages and lymphocytes

**Fig. 5:**
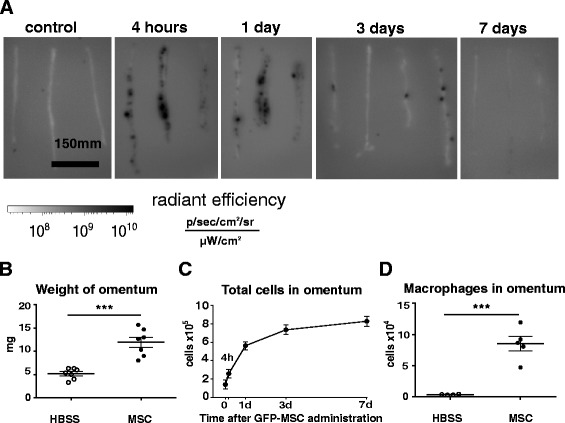
IP injected MSC localized to the omentum and recruit immune cells. **a** Ex vivo fluorescent images of the omenta excised from GFP-MSC-injected BALB/c mice. To help in visualization of the GFP signal in the transparent omentum, reversed logarithmic grayscale was applied to the images. *Black areas* correspond to the GFP-positive signal. Omenta were isolated from MSC- or HBSS-injected animals, weighed, digested and analyzed by flow cytometry. **b** Scatter plots represent weights of individual omenta. **c** Total cells after digestion and **d** total macrophages at 3 days after injections. Error bars are ± SEM. *P < 0.05, **P < 0.01, ***P < 0.001 compared to HBSS. *GFP* green fluorescent protein, *HBSS* Hank’s Balanced Salt Solution, *MSC* mesenchymal stem cells

Since the omentum was a major site for the firm attachment of the aggregates, the organ was excised and examined. Fluorescence assays indicated that the content of GFP-MSC increased sharply by 4 h, decreased by 3 days and were not detected by 7 days (Fig. 5a). As the MSC decreased, there was a marked increase in the weight of the omentum (Fig. 5b), the total cell content (Fig. 5c), and macrophage content (Fig. 5d), observations suggesting recruitment of macrophages and lymphocytes into the omentum that continued after most of the MSC had disappeared (Additional file 2: Figure S1B).

### IP injection of MSC triggers increased expression of murine cytokines and preconditions the immune system

The IP infusion of MSC stimulated increased secretion of a series of mouse cytokines into the peritoneal lavage in a time-dependent manner (Additional file 7: Figure S3). To define the source of the cytokines detected in the lavage after injection of MSC, we used real-time PCR assays of omentum, mesentery, cells from peritoneal lavage, and jejunal lymph nodes (Additional file 7: Figure S3D). The results suggested that the omentum was the major source of the increases in expression of mouse *Il10, Ifng* and *Il13* but both the omentum and the cells in the peritoneal lavage made major contributions to the increase in *Cox2* (cyclooxygenase; prostaglandin-endoperoxide synthase). Human MSCs showed distinct change in their gene expression profiles after administration inside the peritoneal cavity. Real-time PCR assays showed upregulation of human *TGFβ1*, *COX2* and *TSG6* in injected cells in the peritoneal cavity in comparison to unstimulated in vitro cultures of MSCs.

The persistence of changes in the peritoneum suggested that the IP infusion of MSC had pre-conditioned the immune system of the mouse. Therefore, we tested the effects of challenging the mice with the bacterial extract LPS. Mice received an IP injection of MSC followed by IV infusion of LPS 3 days later. Three hours after the LPS injections, the mice were sacrificed and the lavage fluid was collected for cytokine measurements (Additional file 8: Figure S4). Among the early responding cytokines, the preconditioning enhanced the expression of IL-12p70, MCP1, and CXCL1 (Additional file 8: Figure S4A). Among the intermediate responding cytokines, preconditioning enhanced the levels of IL-10 and greatly enhanced the levels of IFNγ and TNFα (Additional file 8: Figure S4B). MSC preconditioning had no effect on the levels of the late responding cytokines PGE_2_ and IL-13 (Additional file 8: Figure S4C). Of special importance was the observation that MSC killed by repeated cycles of freezing and thawing had little effect in preconditioning the immune system, an observation that suggested an active rather than passive role of the IP-delivered MSC.

## Discussion

The results demonstrated that, after human MSC were injected into peritoneum of immune-competent mice, they quickly formed aggregates with the resident macrophages and the B220+ cells of the peritoneal cavity. The aggregation was so rapid that less than 10 % of the MSC were recovered as single cells in the peritoneal lavage after 20 min. At 4 h, about half of the injected MSC were recovered in the peritoneal lavage fluid as aggregates that were readily isolated by low-speed centrifugation. At 1 day, only a few of the aggregates containing MSC were found in the peritoneal lavage. Instead they were found as aggregates attached to multiple sites in the peritoneal cavity. Most of the aggregates were loosely attached but those attached to the omentum and sites within the mesentery were embedded within the tissues. The survival and viability of the human cells was assessed by real-time RT-PCR for mRNA specific for human GAPDH and measuring levels of GFP fluorescence emitted by GFP-MSC. Both signals that reflect live cells indicated that the cells survived transiently and most disappeared after about 3 days.

Surprisingly, less than 1 in 3000 (0.003 %) of the injected cells were recovered in jejunal lymph nodes or the spleen. The results present a contrast to previous observations with IP injections of splenocytes in which most of the cells passed into the lymphatic system and accumulate in lymph nodes [31]. The results therefore indicated that, apparently because of the rapid aggregation, the MSC had limited direct access to lymphatics. However, it is necessary to mention that the human MSC could have also lacked the specific human signals required for migration to lymph nodes or the spleen in the mouse environment.

One interesting observation was that although the number of MSC in the omentum rapidly decreased after 3 days they initiated a cascade whereby large numbers of mouse cells were recruited to the omentum and the organ doubled in weight. The observations are probably explained by the peaks of the proinflammatory mouse cytokines IL-6 and IL-12p70 at 4 h and IFNγ and TNFα at 72 h that probably attracted and activated macrophages.

The rapid aggregation of MSC with macrophages and B220+ cells explains some of the therapeutic effects previously observed with IP administration of MSC in animal models. As reported previously [30], the MSC in the aggregates formed in the peritoneum within 4 h were activated to express high levels of potentially therapeutic human genes such as the genes for the natural anti-inflammatory protein TSG-6, for COX2 that is a critical enzyme in the synthetic pathway for PGE_2_, and for STC-1 that can reduce reactive oxygen species. The activation was more rapid than seen with human MSC cultured as hanging drops under conditions in which the cells condense into spheres, and it is probably accelerated by the presence of the macrophages and B220+ cells in the aggregates. The rapid activation of human MSC injected IP into immunocompetent mice probably explains the effectiveness of this route of administration of human MSC in two models of sterile inflammation: ethanol injury to the cornea [12] and zymosan-induced peritonitis [13]. In the cornea model, the window for effective therapy is less than 6 h [38] and, in the peritonitis model, the beneficial effects were seen within 4 h [13]. In both models, the therapeutic responses were linked to expression of TSG-6, since MSC with a silencing-RNA knockdown of the TSG-6 gene were ineffective and most of the effects of MSC were reproduced by administration of recombinant TSG-6. In the present study we also show the upregulation of TSG-6 in injected MSCs. Similar rapid activations of IP MSC may also explain some of the therapeutic effects observed in other models [8–11]. The MSC used in the reported experiments were either isogeneic or allogeneic but could have invoked the rapid reactions of the peritoneal macrophages and B1 lymphocytes because the MSC acted as foreign bodies in the peritoneum, or because they had acquired foreign antigens during their isolation and expansion in culture. As shown previously [39], MSC rapidly internalize large amounts of fetal bovine proteins from the medium used to culture the cells and the internalized proteins stimulate immune responses after IV infusions of the cells into mice. The isogeneic mouse MSC used by Yousefi et al. [8], Scruggs et al. [9], and Cheng et al. [10] were all isolated and expanded in medium containing high concentrations of FBS. In the Wistar rat model for induced colitis study, Castelo-Branco et al. [11] used bone marrow- and adipose-derived MSC that were isolated and expanded in 15 % or 20 % fetal calf serum. Therefore, either the cells themselves, or the foreign proteins they carried, could have prompted rapid aggregation with peritoneal macrophages and B220+ cells and the subsequent activation of therapeutic genes in the MSC. Sorting out these possibilities is technically challenging because of the tendency of mouse MSC to undergo spontaneous transformation during expansion in culture [40, 41], and the extensive manipulations of MSC in culture required to remove internalized fetal calf proteins [39].

One of the limitations of the present study is that human MSC were employed in immunocompetent mice. Therefore, some of the effects can be ascribed to inflammatory and immune reactions to xenogeneic cells. However, comparable experiments with mouse MSC are limited by the inability of the cells to be expanded significantly without undergoing spontaneous transformation [40, 41]. Another limitation is that the data did not establish whether the MSC were more effective in preconditioning the immune system than cells such as fibroblasts. However, the data demonstrated that three preparations of human MSC isolated and expanded with the same protocols varied in their efficacy. Therefore, it will probably be necessary to compare large numbers of different preparations of MSC and large numbers of control cells to establish which are the most effective. The results did establish that live MSC were far more effective than dead MSC in preconditioning the mice in their response to LPS. Therefore the results were not explained by inflammatory or immune reactions to human cellular proteins or cell debris.

## Conclusions

In conclusion, we demonstrated that IP injected human MSC quickly aggregate and the aggregates attach to the mesentery, omentum and other sites in the peritoneal cavity. In contrast, only small amounts of the cells migrate to the spleen and jejunal lymph nodes. Aggregation and attachment to tissues was accompanied by the recruitment of immune cells and sequential changes in the production of mouse cytokines. The formation of the aggregates probably limits access of the cells to the systemic circulation.

## Additional files

Additional file 1: Table S1. Characteristics of the MSC donor samples used in the current study. (PDF 56 kb) Additional file 2: Figure S1. Detection of MSC in mouse tissues and peritoneal lavage by real-time PCR. (A) Standard curves of known amounts of human cells added to mouse tissues plotted versus corresponding delta Ct values (Ct human GAPDH – Ct values eukaryotic 18S) obtained by real-time PCR in the same tissues. Points represent average values for delta-Ct, lines represent logarithmic model fit. (B) Peritoneal lavage from human MSC-injected BALB/c was collected and total number of recovered cells was enumerated with hemocytometer. Values are mean ± SEM, n = 4–5. *OM* omentum, *MT* mesentery, *PLP* cell pellet from peritoneal lavage, *JLN* jejunal lymph nodes, *SP* spleen. (PDF 58 kb) Additional file 3: Table S2. Goodness of logarithmic fit of standard curves. (PDF 44 kb) Additional file 4: Figure S2. Effects of IP injected MSC to cytokine levels in the mouse serum. Mouse IL10 and IL13 levels in the serum were assayed 72 h after IP injection of MSC. (PDF 18 kb) Additional file 5: Table S3. Biological replicates of Ct values for eukaryotic 18S and human GAPDH assayed by real-time PCR in mouse tissues after MSC administration. (PDF 77 kb) Additional file 6: Video S1. Z-stack of GFP-MSC aggregate in omentum. Omentum from GFP-MSC injected animal was isolated after 24 h post administration, fixed and labeled for B220. Z-stacks with step of 5 μm was obtained and converted into AVI video file using ImageJ software. Human GFP-MSC are seen in the center of the aggregate (green), while B220-positive cells lymphocytes have no specific localization and are seen in the center and also at the periphery of the aggregate. Magnification 20 ×. (AVI 5314 kb) Additional file 7: Figure S3. Production of mouse cytokines in the peritoneal cavity after MSC administration. Mouse cytokines were assayed in peritoneal lavage of MSC-injected BALB/c mice. Based on the timing of the cytokine production, they were categorized into (A) early- (peak production within 4 h), (B) intermediate- (peak production on day 3), or (C) late-responding cytokines (peak production beyond day 3). Data are representative of two independent experiments. Values are arithmetic means ± SEM, n = 4–6. (D) Relative gene expression of selected mouse cytokines in omentum (OM), mesentery (MT), peritoneal lavage pellet (PLP) and jejunal lymph nodes (JLN). Values are average gene expression values normalized to mouse *Gapdh* using 2^–ΔCt^ method ± SEM, n = 4 to 5. **P* < 0.05; ***P* < 0.01; ****P* < 0.001 compared to baseline expression levels. (E) Relative gene expression of human cytokines in omentum, mesentery, peritoneal lavage pellet and jejunal lymph nodes. Values are fold change of gene expression in vivo over corresponding gene expression in unstimulated in vitro cultures of MSCs prior to the injection into mice represented on the graph by dotted line. The expression values were obtained using 2^–ΔΔCt^ method with human GAPDH as housekeeping control. (PDF 105 kb) Additional file 8: Figure S4. Preconditioning of the mouse immune system by IP injected MSC. Live or dead MSC were injected IP into BALB/c mice 3 days prior to systemic administration of LPS. Identical experiments without LPS administration were performed in naïve mice. Mouse cytokines in the peritoneal lavage were measured at 3 days after MSC administration and 3 h after LPS administration. Based on the timing of the cytokine production, they were categorized into (A) early- (peak production within 4 h), (B) intermediate- (peak production on day 3), or (C) late-responding cytokines (peak production beyond day 3). Values represent mean ± SEM, n = 4–7. **P* < 0.05; ***P* < 0.01; ****P* < 0.001 compared to vehicle controls in each experimental setting. The Y-axis is logarithmically transformed. (PDF 62 kb)

## Abbreviations

αMEM: Alpha minimum essential medium alpha
ANOVA: Analysis of variance
BALB/c: Bagg Albino (inbred research mouse strain)
BSA: Bovine serum albumin
CCM: Complete culture medium
cDNA: Complementary deoxyribonucleic acid
COX2: Cyclooxygenase-2
Ct: Critical threshold
CXCL1: Chemokine (C-X-C motif) ligand 1
DAPI: 4′,6-Diamidino-2-phenylindole
DNAse: Deoxyribonuclease
EDTA: Ethylenediaminetetraacetic acid
ELISA: Enzyme-linked immunosorbent assay
FBS: Fetal bovine serum
GAPDH: Glyceraldehyde 3-phosphate dehydrogenase
GFP: Green fluorescent protein
HBSS: Hank’s Balanced Salt Solution
IFN: interferon
Ig: Immunoglobulin
IL: Interleukin
IP: Intraperitoneal
IV: Intravenous
LPS: Lipopolysaccharide
MCP1: Monocyte chemoattractant protein-1
mRNA: Messenger ribonucleic acid
MSC: Mesenchymal stem cells
NIH: National Institutes of Health
OCT: Optimal cutting temperature compound
PBS: Phosphate-buffered saline
PCR: Polymerase chain reaction
PG: Prostaglandin
PTGS2: Prostaglandin-endoperoxide synthase 2
RNA: Ribonucleic acid
RT: Reverse transcription
STC-1: Stanniocalcin-1
TBST: Tris-buffered saline and tween 20
TGF: Transforming growth factor
Th: T-helper
TNF: Tumor necrosis factor
TSG-6: Tumor necrosis factor-inducible gene 6 protein
