# Supplementary material for: Intraperitoneally infused human mesenchymal stem cells form aggregates with mouse immune cells and attach to peritoneal organs
Source: Stem Cell Res Ther. 2016 Feb 10;7:27. doi: 10.1186/s13287-016-0284-5 (PMC4748482; doi:10.1186/s13287-016-0284-5)
Supplement: Additional file 1: Table S1. — Characteristics of the MSC donor samples used in the current study. (PDF 56 kb) [file 13287_2016_284_MOESM1_ESM.pdf]

**Supplemental Table 1**. Characteristics of the MSC donor samples used in the current study.

| **Donor**[a] (ID#/gender /age) | **P0 Yield**[b] (x $10^6$)/ days | **P0→P1 Yield (Master Bank)**[c] (plating cells per $cm^2$ / harvest cells per $cm^2$ /days) | **P1 Cumulative PDs**[d] | **P1→P2 Yield (Working Bank)** (plating cells per $cm^2$ /harvest cells per $cm^2$ /days) | **P2 Cumulative PDs** | **P2 Diff.**[e] (osteo/ adipo/ CFU) | **P2 Epitopes**[f] (+/-) |
|---|---|---|---|---|---|---|---|
| 7032R/M/26 | 0.8/6 | 50/14557/8 | j+8.3 | 50/6000/6 | j+6.8 | 3/3/49 | >95/<3 |
| 7068L/M/37 | 1.17/5 | 50/7595/8 | j+7.2 | 100/7546/7 | j+13.4 | 3/4/45 | >95/<3 |
| 7075L/M/24 | 2.96/6 | 100/6329/7 | j+6.0 | 100/9287/7 | j+12.5 | 4/1/49 | >95/<3 |

[a]Donors identified by anonymous number and sampling of marrow from left (L) or right (R) iliac crest/gender/age.
[b]P0 yield defined as number of cells obtained by plating mononuclear cells from ficoll gradient at high density (6,000 to 30,000 per $cm^2$)/ days cells were incubated.
[c]Master Bank (P1 cells) defined by number of cells plated per $cm^2$/number of cells harvested per $cm^2$/days of incubation.
[d]Cumulative population doublings defined as j + observed value because number of population doublings required to generate P0 cells cannot be estimated. PD calculated as $2^n$ = fold-increase or n = $\log_{10}$[fold-increase]/0.301.
[e]P2 Diff. assayed by culture of aliquots in differentiation conditions and visually scoring on +1 to +4 scale after staining with Alizarin Red or Oil-Red-O. CFUs assayed by plating at very low density to obtain % cells that generate colonies in 2 weeks.
[f]Positive epitopes (CD-29, -44, -49c, -59, -90, -105, -147 and -166) and negative epitopes (CD-34, -36, -45, and -117)
