# Supplementary figures and images for: Intraperitoneally infused human mesenchymal stem cells form aggregates with mouse immune cells and attach to peritoneal organs
Source: Stem Cell Res Ther. 2016 Feb 10;7:27. doi: 10.1186/s13287-016-0284-5 (PMC4748482; doi:10.1186/s13287-016-0284-5)

**A****PCR Standard Curves**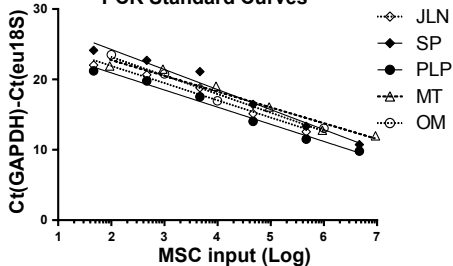**B****Recovered cells from peritoneal cavity**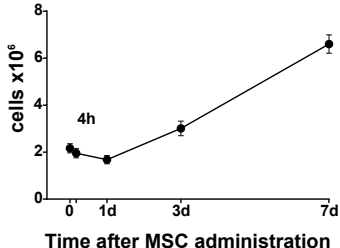

Supplement: Additional file 2: Figure S1. — Detection of MSC in mouse tissues and peritoneal lavage by real-time PCR. (A) Standard curves of known amounts of human cells added to mouse tissues plotted versus corresponding delta Ct values (Ct human GAPDH – Ct values eukaryotic 18S) obtained by real-time PCR in the same tissues. Points represent average values for delta-Ct, lines represent logarithmic model fit. (B) Peritoneal lavage from human MSC-injected BALB/c was collected and total number of recovered cells was enumerated with hemocytometer. Values are mean ± SEM, n = 4–5. OM omentum, MT mesentery, PLP cell pellet from peritoneal lavage, JLN jejunal lymph nodes, SP spleen. (PDF 58 kb) [file 13287_2016_284_MOESM2_ESM.pdf]

# Systemic response (serum)

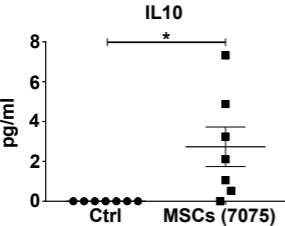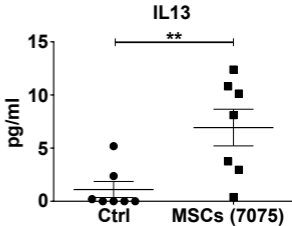

Supplement: Additional file 4: Figure S2. — Effects of IP injected MSC to cytokine levels in the mouse serum. Mouse IL10 and IL13 levels in the serum were assayed 72 h after IP injection of MSC. (PDF 18 kb) [file 13287_2016_284_MOESM4_ESM.pdf]

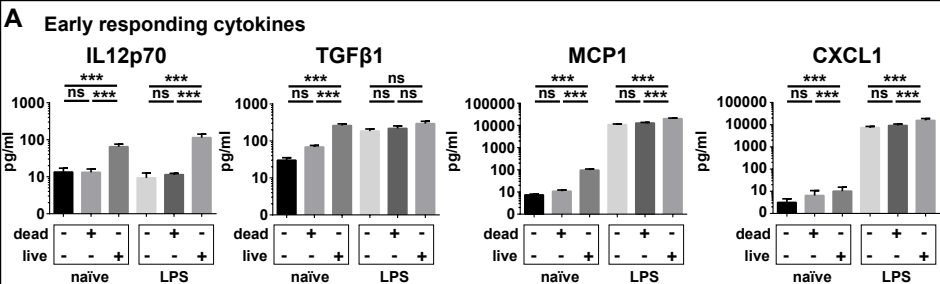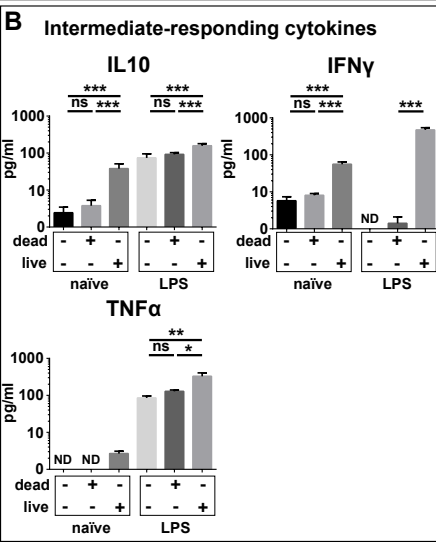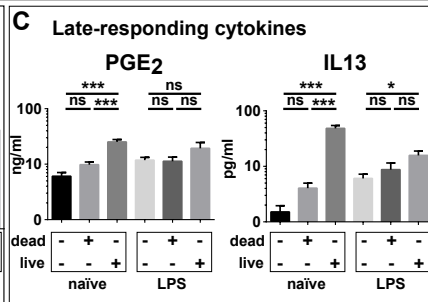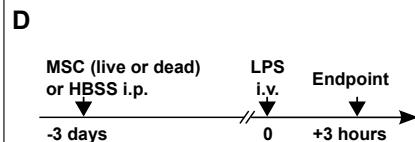

Supplement: Additional file 8: Figure S4. — Preconditioning of the mouse immune system by IP injected MSC. Live or dead MSC were injected IP into BALB/c mice 3 days prior to systemic administration of LPS. Identical experiments without LPS administration were performed in naïve mice. Mouse cytokines in the peritoneal lavage were measured at 3 days after MSC administration and 3 h after LPS administration. Based on the timing of the cytokine production, they were categorized into (A) early- (peak production within 4 h), (B) intermediate- (peak production on day 3), or (C) late-responding cytokines (peak production beyond day 3). Values represent mean ± SEM, n = 4–7. *P < 0.05; **P < 0.01; ***P < 0.001 compared to vehicle controls in each experimental setting. The Y-axis is logarithmically transformed. (PDF 62 kb) [file 13287_2016_284_MOESM8_ESM.pdf]
