# Supplementary material for: Intraperitoneally infused human mesenchymal stem cells form aggregates with mouse immune cells and attach to peritoneal organs
Source: Stem Cell Res Ther. 2016 Feb 10;7:27. doi: 10.1186/s13287-016-0284-5 (PMC4748482; doi:10.1186/s13287-016-0284-5)
Supplement: Additional file 3: Table S2. — Goodness of logarithmic fit of standard curves. (PDF 44 kb) [file 13287_2016_284_MOESM3_ESM.pdf]

Supplemental table 2 Goodness of logarythmic fit of standard curves.

Tissue	r^2	Formula
JLN	0.96	$f(x)=-0.88\ln(x)+25.20$
SP	0.97	$f(x)=-1.24\ln(x)+29.97$
PLP	0.96	$f(x)=-0.93\ln(x)+24.37$
MT	0.96	$f(x)=-0.98\ln(x)+27.25$
OM	0.99	$f(x)=-1.21\ln(x)+29.11$

Abbreviations: OM – omentum, MT – mesentery, PLP – cell pellet from peritoneal lavage, JLN - jejunal lymph nodes, SP – spleen.
