# Supplementary material for: Intraperitoneally infused human mesenchymal stem cells form aggregates with mouse immune cells and attach to peritoneal organs
Source: Stem Cell Res Ther. 2016 Feb 10;7:27. doi: 10.1186/s13287-016-0284-5 (PMC4748482; doi:10.1186/s13287-016-0284-5)
Supplement: Additional file 5: Table S3. — Biological replicates of Ct values for eukaryotic 18S and human GAPDH assayed by real-time PCR in mouse tissues after MSC administration. (PDF 77 kb) [file 13287_2016_284_MOESM5_ESM.pdf]

Supplemental Table 3. Biological replicates of Ct values for eukariotic 18S and human GAPDH assayed by real-time PCR in mouse tissues after MSC administration

Tissue:		Jejunal lymph nodes		Spleen		Lavage pellet		Mesentery		Omentum	
Time[a]	Replicate[b]	Ct eu18S	Ct GAPDH	Ct eu18S	Ct GAPDH	Ct eu18S	Ct GAPDH	Ct eu18S	Ct GAPDH	Ct eu18S	Ct GAPDH
**0 h**	1	10.11	ND	10.57	ND	10.13	ND	9.85	ND	8.14	ND
	2	10.16	ND	11.2	ND	10.8	ND	9.8	ND	8.41	ND
	3	9.95	ND	11.34	ND	10.98	ND	9.71	ND	7.9	ND
	4	10.26	ND	10.52	ND	11.35	ND	9.91	ND	7.43	ND
**4 h**	1	10.53	34.25	10.68	29.05	11.05	21.13	9.93	34.94	6.89	21.04
	2	9.89	29.5	11.2	28.64	11.43	22.16	–	–	7.28	20.84
	3	10.24	30.68	10.82	30.71	10.92	22.25	10.18	26.45	7.61	22.33
	4	–	–	11.02	31.05	10.97	23.03	10.13	24.4	7.88	22.87
	5	10.66	32.22	9.99	30.68	10.57	22.28	10.14	28	6.91	25.74
**24 h**	1	9.81	ND	9.85	30.03	10.76	24.53	11.71	38.55	7.08	20.41
	2	10.22	29.92	10.57	29.58	11.26	30.12	11.73	25.43	6.8	22.34
	3	10.27	38.61	9.81	34.17	10.83	28.76	11.24	27.04	7.15	23.8
	4	10.69	39.82	10.77	31.24	11.13	26.17	10.5	25.62	6.66	21.1
	5	10.48	33.69	10.72	31.12	11.42	24.6	10.53	25.46	7.38	21.25
**72 h**	1	10.02	ND	10.85	39.63	10.64	38.69	10.58	31.53	8.8	31.46
	2	10.43	ND	10.88	36.84	10.61	31.89	9.86	30.07	10.09	25.78
	3	9.85	37.59	10.21	34.57	–	–	9.87	28.84	10.41	26.44
	4	9.87	ND	10.85	33.78	–	–	9.96	29.21	8.45	29.14
	5	9.39	ND	10.26	35.58	10.5	35.43	9.91	28.9	8.63	25.22
**168 h**	1	10.58	ND	10.16	ND	12.68	ND	9.8	ND	10.28	ND
	2	10.6	ND	10.85	ND	13.16	ND	11.15	ND	9.52	ND
	3	10.32	39.87	10.09	ND	13.97	ND	11.47	ND	9.53	ND
	4	9.76	ND	10.88	ND	13.22	ND	11.13	ND	8.97	ND

[a] Time (hours)after the administration of human MSC into peritoneal cavity of mice.

[b]Biological replicates of BALB/c mice, injected IP with MSC

Abbreviations: Ct – real-time PCR critical threshold values for target genes; ND – not detected, horizontal line – not used; eu18S – eukaryotic 18S ribosomal RNA; GAPDH - Glyceraldehyde 3-phosphate dehydrogenase.
