# Supplementary material for: Intraperitoneally infused human mesenchymal stem cells form aggregates with mouse immune cells and attach to peritoneal organs
Source: Stem Cell Res Ther. 2016 Feb 10;7:27. doi: 10.1186/s13287-016-0284-5 (PMC4748482; doi:10.1186/s13287-016-0284-5)
Supplement: Additional file 7: Figure S3. — Production of mouse cytokines in the peritoneal cavity after MSC administration. Mouse cytokines were assayed in peritoneal lavage of MSC-injected BALB/c mice. Based on the timing of the cytokine production, they were categorized into (A) early- (peak production within 4 h), (B) intermediate- (peak production on day 3), or (C) late-responding cytokines (peak production beyond day 3). Data are representative of two independent experiments. Values are arithmetic means ± SEM, n = 4–6. (D) Relative gene expression of selected mouse cytokines in omentum (OM), mesentery (MT), peritoneal lavage pellet (PLP) and jejunal lymph nodes (JLN). Values are average gene expression values normalized to mouse Gapdh using 2–ΔCt method ± SEM, n = 4 to 5. *P < 0.05; **P < 0.01; ***P < 0.001 compared to baseline expression levels. (E) Relative gene expression of human cytokines in omentum, mesentery, peritoneal lavage pellet and jejunal lymph nodes. Values are fold change of gene expression in vivo over corresponding gene expression in unstimulated in vitro cultures of MSCs prior to the injection into mice represented on the graph by dotted line. The expression values were obtained using 2–ΔΔCt method with human GAPDH as housekeeping control. (PDF 105 kb) [file 13287_2016_284_MOESM7_ESM.pdf]

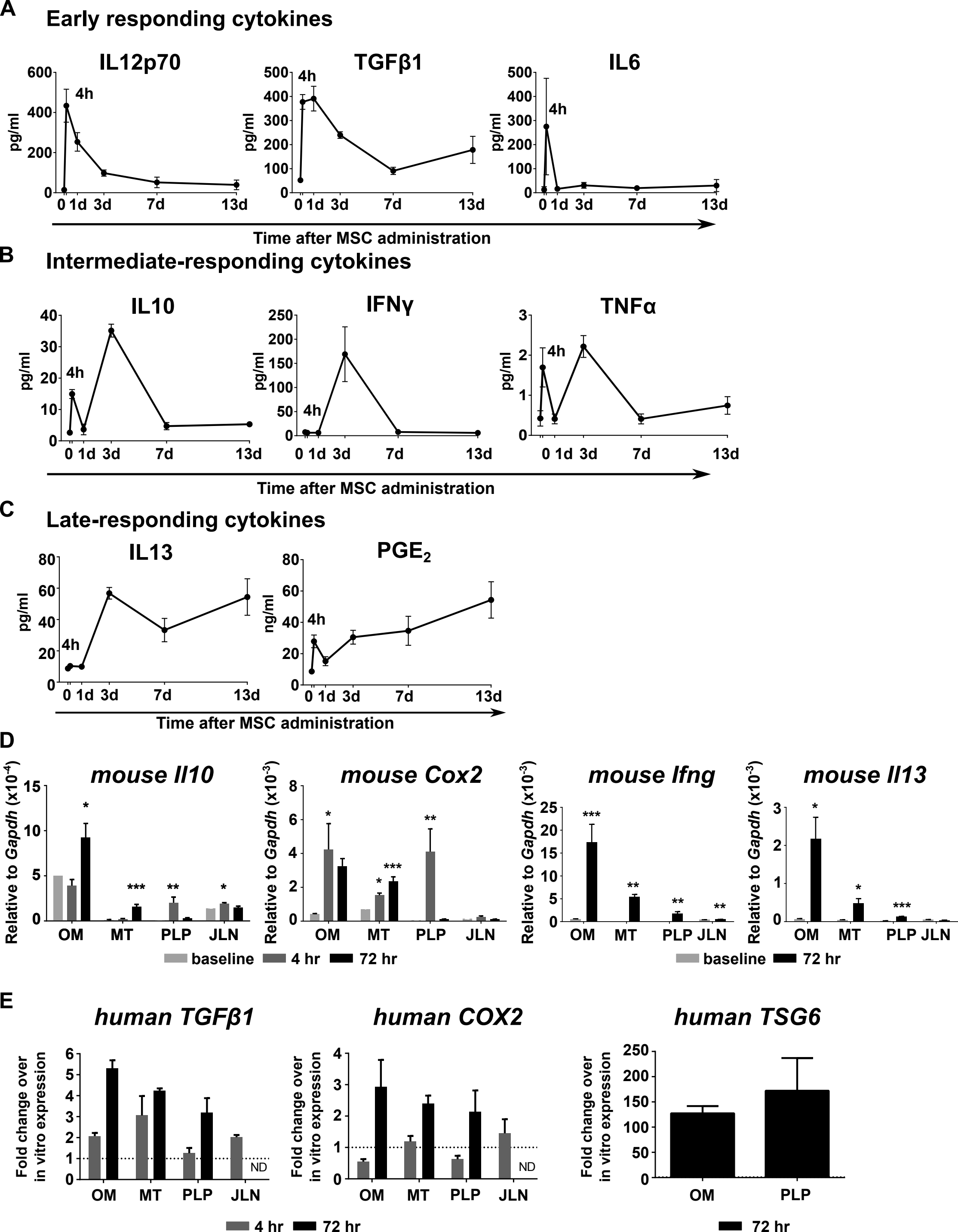
A
Early responding cytokines
IL12p70
TGFβ1
IL6
pg/ml
4h
0 1d 3d 7d 13d
Time after MSC administration
B
Intermediate-responding cytokines
IL10
IFNγ
TNFα
pg/ml
4h
0 1d 3d 7d 13d
Time after MSC administration
C
Late-responding cytokines
IL13
PGE2
pg/ml
ng/ml
4h
0 1d 3d 7d 13d
Time after MSC administration
D
mouse Il10
mouse Cox2
mouse Ifng
mouse Il13
Relative to Gapdh (x10-4)
Relative to Gapdh (x10-3)
OM MT PLP JLN
baseline 4 hr 72 hr
baseline 72 hr
E
human TGFβ1
human COX2
human TSG6
Fold change over in vitro expression
ND
OM MT PLP JLN
OM PLP
4 hr 72 hr
72 hr
